# Measuring Therapeutic Alliance for Tobacco Cessation Counseling for Behavioral Health Clinicians

**DOI:** 10.1155/2021/6671899

**Published:** 2021-03-13

**Authors:** Bruce Christiansen, Stevens S. Smith, Michael C. Fiore

**Affiliations:** Center for Tobacco Research and Intervention, School of Medicine and Public Health, University of Wisconsin-Madison, 1930 Monroe St., Madison, WI 53711, USA

## Abstract

**Introduction:**

Those coping with significant mental illness smoke at a high prevalence rate. Increasingly, behavioral health clinicians (BHCs) are being asked to provide tobacco-dependence interventions. In this context, it is important to measure their success at doing so. While the Working Alliance Inventory (WAI) is a well-established measurement of the effectiveness of therapeutic alliance, it is not specific to tobacco-dependence interventions. The Working Alliance Inventory for Tobacco (WAIT-3) has been found valid for tobacco cessation counselors (health providers who address tobacco), but its validity has not been established when BHCs address tobacco cessation as part of addressing all other needs of their patients. The purpose of this study was to examine the validity of the WAIT-3 in the context of behavioral health clinicians.

**Methods:**

Wisconsin Community Support Programs and Comprehensive Community Services programs distributed an anonymous, brief (14 items) survey to 1,930 of their clients. Measured variables included smoking status, behavioral intentions regarding quitting, and perception of help received from their clinic. Respondents could enter a chance to win a gift card as a thank you.

**Results:**

WAIT-3 scores were correlated with quitting-related variables. Compared to those with lower WAIT-3 scores, those with higher scores reported more attempts to quit, were more motivated to quit, were more likely to have a smoking cessation/reduction goal in their general treatment plan, had more conversations about quitting with their BHC, and wanted more help from their BHC to quit.

**Conclusions:**

The WAIT-3 may be a valid way to measure the effectiveness of BHCs to address the tobacco use of their patients. Next steps include establishing its predictive validity.

## 1. Introduction

Smoking prevalence is elevated among those coping with a mental illness. Among those reporting serious psychological distress, the prevalence of smoking is 37.2%, two and a half times the rate among those not reporting serious psychological stress (14.0%) [1]. Yet, such individuals often do not receive evidence-based tobacco-dependence treatment. Nationally, only 48.9% of mental health treatment facilities screen for tobacco use, and only 37.6% provide smoking cessation counseling [2]. Thus, there is a great need to increase the provision of cessation treatment to this population.

In Wisconsin, those coping with a significant and persistent mental illness receive care from Community Support Programs (CPS) and Comprehensive Community Services (CCS) programs. The 62 CSPs provide wraparound services to 5,559 individuals in Wisconsin with diagnoses such as schizophrenia, bipolar disorder, and major affective disorder who have a history of acute treatment services and need support in major areas of community living [3]. The 67 CCSs provide care to 8,897 individuals in Wisconsin who have functional impairment in one or more major life activities and who need less intense services than those in CSP but more service than is typically available in outpatient treatment [4].

As behavioral health clinicians (BHCs) are asked to provide tobacco-dependence interventions, it is important to assess their ability to do so. The Working Alliance Inventory (WAI) is a well-established measurement of therapeutic alliance. It correlates with therapy outcome and focuses on counselor-client agreement on goals, agreement on method of achieving goals, and the bond between counselor and client [5–7]. However, the WAI assesses general therapeutic alliance rather than therapeutic alliance specific to smoking cessation interventions. As such, it would not suffice as a measure of tobacco counseling effectiveness when used by BHCs who are helping clients with multiple goals through a general therapeutic relationship.

Warlick et al. modified the WAI to make it specific to tobacco cessation counseling [8]. They found that both the 3- and 12-item Working Alliance Inventory for Tobacco (WAIT-3 and WAIT-12) had acceptable psychometric properties and validity within a sample of smokers, recruited over the internet, when asked about their “smoking cessation counselor,” defined as a health care provider or professional who talked with them about quitting smoking. Unknown is if the WAIT is valid when used by BHCs who are helping patients address tobacco use in the context of helping them with other mental health needs. The purpose of this study was to examine the validity of the WAIT-3 in a sample of smokers receiving tobacco cessation help from their BHC.

## 2. Methods

A 14-item tobacco survey was sent to each of Wisconsin's CSPs and CCSs. Each CSP/CCS was sent a number of surveys sufficient for 15% of their adult clients (1,930). The CSP/CCSs were asked to distribute the surveys without concern to who received them, collect them, and mail them back. The survey was anonymous with no client identifiers. Clients were given the opportunity to enter their name separately in a drawing to win one of six $25.00 gift cards. The survey was conducted as part of evaluating a tobacco training program for CSP/CCS clinicians. As program evaluation, it was deemed exempt from IRB review. Data were collected between 12-1-19 and 3-15-20.

The survey asked for smoking status, behavioral intention regarding addressing tobacco use, desire and need to quit, recent attempts to quit or reduce smoking, past receipt of help to quit by their clinic, and opinions/attitudes/beliefs about getting help from their clinic and the clinic role in addressing their tobacco use. For behavioral intention, respondents were asked to indicate which of four intentions best described them: willing to make a quit attempt in the near future; not willing to quit but willing to take action to learn how to quit or to reduce; not willing to take action but willing to talk about their use of tobacco; or not even willing to talk about it at the present time. The opinion/attitude/belief questions were asked on a 5-point Likert scale from strongly disagree to strongly agree. Example items were: “I want my CSP/CCS to help me quit using tobacco”; “My tobacco use is of no concern to my CSP/CCS”; and “I want to quit, but don't think I can.”

The survey also included the WAIT-3 items. These items were intended to measure three dimensions of therapeutic alliance: bond between client and counselor, goals related to tobacco cessation, and methods for achieving the goals. The original WAIT-3 item that measures bond (“I felt my tobacco counselor appreciated me.)” was modified to make it specific to a bond about tobacco-dependence treatment so as not to measure therapeutic bond in general. Survey respondents were asked “Conversations with CSP/CCS staff over the last six months show me that staff: (1) appreciate my point of view on tobacco use [bond item]; (2) agree on clear tobacco treatment goals for me [goal item]; and (3) agree on a method I will use to achieve my tobacco goals [method item].” To respond, smokers selected, “seldom, sometimes, fairly often, very often, or always” coded 1-5, respectively, for scoring.

A WAIT-3 score was calculated for each respondent that had a range of 3 to 15 by summing across the three items. This WAIT-3 score was then studied relative to self-reported smoking behaviors indicative of efforts to quit.

## 3. Results

Of the 1930 surveys distributed to the CSPs/CCSs, 782 were returned, for a response rate of 40.5%. Of the returned surveys, 47.1% were from CSP clients and 52.9% were from CCS clients. Of these, 424 (55.9%) were returned from current smokers (60.9% of CSP clients and 51.5% of CCS clients). Of these 424, 263 (62.0%) indicated that they had had conversations with the CSP/CCS staff about tobacco in the past six months and thus answered the WAIT-3 questions. These 263 surveys were used in the remaining analyses.

**Figure 1:**
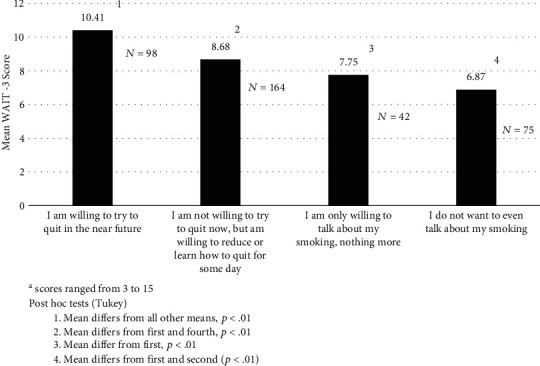
Mean WAIT-3 score and behavioral intention (scores ranged from 3 to 15). Post hoc tests (Tukey). (1) Mean differs from all other means, *p* < .01. (2) Mean differs from first and fourth, *p* < .01. (3) Mean differs from first, *p* < .01. (4) Mean differs from first and second (*p* < .01).

The WAIT-3 score ranged from 3 to 15 with a mean of 8.84 and a standard deviation of 3.54. Cronbach's *α* for the WAIT-3 was .85. A Principal component factor analysis extracted a single factor (eigenvalue > 1) that accounted for 80.8% of the variance. The bond item loaded .84 on this factor, the goal item, .93, and the method item, .93. There was no difference between the mean scores of CSP clients and CCS clients. (8.94 (3.39SD) vs. 8.90 (3.75 SD), respectively, *t* = .074, df = 254, *p* = .94). Those who tried to quit in the past 6 months had a higher WAIT-3 score than those who did not (9.4 vs. 8.1, respectively, *t* = 2.80, df = 236, *p* < .01) as did those who reduced their smoking compared to those who did not (9.1 vs. 8.1, respectively, *t* = 1.97, *p* = .05). WAIT-3 score differed as a function of the behavioral intention of the smokers (*F* = 10.58, df = 3, *p* < .01). Post hoc tests (Tukey) revealed differences among the means. Smokers who were ready to make a quit attempt had the highest WAIT-3 (10.41) score while those who did not even want to talk about their tobacco use had the lowest (6.87) (see Figure 1). Survey respondents who reported that their overall treatment plan contained a smoking cessation or reduction goal scored higher than those who did not report such a goal (9.9 vs. 8.1, *t* = 4.17, df = 257, *p* < .01). Respondents were asked how often clinic staff talked with them about their smoking. Those who had more conversations had higher WAIT-3 scores (*F* = 10.96, df = 3, *p* < .01). Post hoc tests revealed that those who had a conversation only once in a while had lower mean WAIT-3 scores than those who spoke about smoking often or at almost every visit (7.83 vs. 10.03 and 10.58, respectively, *p* < .01). WAIT-3 scores were related to how much help survey respondents wanted about their smoking (*F* = 5.55, df = 2, *p* < .01). Post hoc testing revealed that those who wanted more help and those who thought the help they received was just about right had significantly higher mean WAIT-3 scores than those who wanted less help (9.52 and 8.88 vs. 6.00, respectively, *p* < .01). Finally, there were significant correlations between WAIT-3 scores and strength of agreement with 10 of 11 beliefs/attitudes/opinions. For example, those who desired to quit had higher WAIT-3 scores (*r* = .34, *p* < .01) as did those who desired the help of their CSP/CCS to quit (*r* = .30, *p* < .01). On the other hand, those who were not at all ready to quit had lower WAIT-3 scores (*r* = −.34, *p* < .01) (see Table 1). There was also a significant correlation of .43 (*p* < .01) between WAIT-3 score and total belief/attitude/opinion score (with five items reverse scored).

## 4. Discussion

As BHCs are increasingly expected to provide evidence-based tobacco-dependence treatment, it will be important to measure their effectiveness in doing so, including the degree to which they form a strong therapeutic alliance around smoking cessation counseling. The general assessment of therapeutic alliance is inappropriate for this purpose because it is not specific to tobacco cessation interventions when delivered by clinicians who are also providing general psychotherapy for other patient needs. The WAIT-3 has supportive validity evidence for use with tobacco counselor health care providers who address tobacco) [8]. The current study suggests that the WAIT-3 may also have validity for general BHCs who address tobacco in the context of treating other needs. It had favorable psychometric properties in this sample including good internal consistency (Cronbach's *α* = .85) with all three items strongly loading on a single factor. Further, the WAIT-3 appears to have convergent validity in this setting with WAIT-3 scores significantly related to client reports of making recent quit attempts, reducing their smoking, behavioral intention regarding addressing tobacco, their desire for help in quitting, the number of discussions about tobacco they had with their clinician, and a host of other attitudes and beliefs favorable to quitting. Further research investigating the use of the WAIT-3 to assess behavioral health clinicians as they provide smoking cessation interventions is warranted.

The WAIT-3 mean score obtained in this study of behavioral health clinicians appear higher than the mean score from two samples of smokers recruited over the internet regarding their “smoking cessation counselor,” defined as a health care provider or professional who talked with them about quitting smoking [8], (8.84 vs. 3.31 and 3.13, respectively). It seems unlikely that this difference reflects word changes made to one of the three items. Perhaps this study measured greater therapeutic alliance because there was likely a longer therapeutic relationship between behavioral health clinicians and smokers in this study than between the more heterogeneous and less-specific “health care provider” and smokers in the other two samples. Cronbach's *α* in this study (.85) was quite similar to those in the other two samples (.88 and .92) suggesting a similar level of internal consistency across diverse contexts.

This study has a number of limitations. First, even though the CSP/CCSs were asked to distribute the survey without regard to who received them, the sample was not necessarily random. Second, the 40.5% response rate was relatively low. For these reasons, results may not reflect accurately the total population of individuals with persistent mental illness. Third, all data were self-report. However, it seems likely that self-reporting being a smoker is accurate because 97.5% of the smokers reported that their CSP/CCS knew their smoking status and the survey was administered by the CSP/CCS. Fourth, all the data were correlational. While it is possible that having strong alliance with therapists about tobacco leads to progress toward quitting, it is also possible that smokers motivated to quit have more favorable perceptions about the tobacco alliance with their therapists. More research, especially temporal and predictive validity data, are needed to explore causal direction.

Survey respondents reflected a population experiencing substantial mental health challenges and significant mental illness. Their very high smoking prevalence (56%) is substantially greater than national surveys of smoking prevalence among those with serious psychological stress (37.2%) [1] and is consistent with findings that smoking prevalence increases with increased severity of mental illness [9, 10]. In this sample, 38% of smokers had never had a conversation about tobacco with their CSP/CCS therapist. This underscores both the need to train behavioral health therapists to deliver evidence-based tobacco-dependence interventions and to assess their skill in doing so. The WAIT-3 might be one way to quickly and easily assess tobacco intervention skills. However, it is unlikely to be sufficient as the only measurement of skill. Other measures such as chart reviews or surveillance of electronic health records for smoking status and documentation of tobacco interventions appropriate to smokers' behavioral intention to quit would be helpful. Perhaps the WAIT-3 could be used as a screening tool to identify therapists with low scores and trigger a more in-depth review.

## 5. Conclusion

Increasingly, behavioral health clinicians are asked to treat tobacco dependence as part of general mental health care. In this context, it is important to have a valid measurement of clinician effectiveness that is specific to providing tobacco-dependence treatment. This study suggests that the 3-item Working Alliance Inventory for Tobacco (WAIT-3) has both favorable psychometric properties and concurrent validity. Next steps include assessing its predictive validity for this purpose.

## Figures and Tables

**Table 1:** Correlation between WAIT-3 scores and attitudes/beliefs/opinions.

Belief/attitude/opinion	Correlation	Significance
It is important for me to quit using tobacco.	.250	<.01
It is not a good idea for a person who is coping with a mental illness to try to quit using tobacco until in full recovery.	-.038	NS
My tobacco use is no concern of my CSP/CCS.	-.246	<.01
I want my CSP/CCS to help me to quit using tobacco.	.298	<.01
When my CSP/CCS addresses my tobacco use, I know that they care about the whole me.	.292	<.01
I want to quit using tobacco.	.335	<.01
It's OK for my CSP/CCS to help me quit using tobacco as long as doing so does not interfere with my other treatment goals.	.266	<.01
I know I need help to quit using tobacco.	.160	.01
I did not come to this CSP/CCS to quit using tobacco so staff should not address my tobacco use.	-.248	<.01
I want to quit using tobacco but do not think I can.	-.124	<.05
I'm not at all ready to quit using tobacco.	-.338	<.01

## Data Availability

Source data can be obtained by contacting the first author.
